# Understanding multi-azole resistance in *Aspergillus fumigatus*: the impact of non-*cyp51A* mutation and efflux pump overexpression

**DOI:** 10.3389/ffunb.2026.1830942

**Published:** 2026-05-28

**Authors:** Pooja Sen, Lokesh Kumar, Jata Shankar, Pooja Vijayaraghavan

**Affiliations:** 1 Amity Institute of Biotechnology, Amity University Uttar Pradesh, Noida, Uttar Pradesh, India; 2 Department of Biotechnology and Bioinformatics, Jaypee University of Information Technology, Solan, India

**Keywords:** *Aspergillus fumigatus*, azole resistance, *cyp51A* mutations, efflux pumps, *hmg1* gene

## Abstract

**Introduction:**

Azole resistance in *Aspergillus fumigatus* is an emerging global concern, significantly limiting treatment options for aspergillosis. The present study aimed to investigate azole resistance mechanisms, focusing on non-*cyp51A* mutations and efflux pump genes overexpression in environmental isolates of *A. fumigatus*.

**Methods:**

A total of 156 soil samples collected from 13 states across India were screened for azole-resistant *A. fumigatus* isolates. Antifungal susceptibility testing was performed against itraconazole, voriconazole, and posaconazole. Molecular characterization included sequencing of the *cyp51A* and *hmg1* genes. Quantitative real-time PCR (qRT-PCR) was conducted to evaluate the expression levels of *cyp51A*, *hmg1*, and efflux pump genes (*mdr1, mdr4, mfsC,* and *cdr1B*).

**Results:**

Nine azole-resistant *A. fumigatus* isolates were identified. Most resistant isolates exhibited cross-resistance to ITC, VRC, and POS. The *cyp51A* gene sequencing revealed known mutations, TR46/T289A, alongside novel mutations, including L77W, L477I, A189P, and W223G. Additionally, 18 non-synonymous mutations were discovered in the *hmg1* gene, of which 14 were located outside the sterol-sensing domain of Hmg1. In contrast, four mutations (F399V, T410P, G385D, and F362Y) were detected within the SSD region, suggesting its potential role in azole resistance. Furthermore, qRT-PCR analysis revealed upregulation of the *cyp51A* gene in all resistant isolates, with selective upregulation of efflux pump genes (*mdr1, mdr4, mfsC*, and *cdr1B*) in various isolates, indicating their probable contribution to resistance.

**Conclusion:**

Our findings highlight the complexity of azole resistance mechanisms, suggesting that non-*cyp51A* mutations and efflux pump overexpression are potentially associated with resistance phenotypes. This study emphasizes the need for further functional validation of these resistance mechanisms and novel therapeutic strategies against azole-resistant *A. fumigatus*.

## 1 Introduction

*Aspergillus fumigatus* is a highly prevalent saprophytic fungus known for producing airborne conidia (Latgé and Chamilos, 2019). Infection occurs primarily through the inhalation of these conidia, leading to a spectrum of clinical conditions collectively termed aspergillosis (Sen et al., 2024), which can range from mild to life-threatening, depending largely on the patient’s immune status (Garcia-Rubio et al., 2017; van de Veerdonk et al., 2017). The mortality rate varies with the severity of the disease; in the most severe form of invasive aspergillosis (IA), mortality can reach up to 90% in certain patient populations (Cadena et al., 2016). Treatment options for aspergillosis are currently limited to three classes of antifungal drugs: azoles, echinocandins, and polyenes. Among these, triazole drugs are the first-line therapy and prophylaxis for IA and other *Aspergillus-*related lung diseases (Denning et al., 2016; Walsh et al., 2008). However, the global rise in azole-resistant *A. fumigatus* (ARAF) strains is diminishing therapeutic options for patients (Chowdhary et al., 2017; Wiederhold and Verweij, 2020).

Data analyzed from a study conducted in the Netherlands indicate that the prevalence of ARAF increased from 0.79% between 1996–2001 to 11.30% in 2013–2018 (Buil et al., 2019; Lestrade et al., 2020). Additionally, a study across 21 research centers worldwide reported triazole-resistant *A. fumigatus* incidence rates ranging from 3.2% to 26.1% (van der Linden et al., 2015; Yang et al., 2021). This growing resistance has contributed to increased treatment failures and higher healthcare costs, presenting a critical public health challenge on a global scale.

With the exception of Antarctica, azole-resistant *A. fumigatus* has been reported worldwide (Burks et al., 2021). ARAF strains can develop through two primary pathways: the medical route, where resistance emerges in patients undergoing long-term azole therapy, leading to the selection of resistant isolates within the host (Mortensen et al., 2011); and the environmental route, where resistance is acquired through the use of demethylation inhibitors (DMIs) in agriculture; while effective against plant pathogens, these fungicides promote cross-resistance in *A. fumigatus* due to their structural similarity to medical triazoles (Garcia-Rubio et al., 2021; Jeanvoine et al., 2020). The primary mechanism of azole resistance in *A. fumigatus* involves alterations in the sterol biosynthesis pathway, typically caused by point mutations in the *cyp51A* gene or the insertion of tandem repeats (TR) in its promoter region, such as TR34/L98H and TR46/Y121F/T289A. However, recent studies have identified ARAF strains that exhibit resistance to all clinical azole drugs without any modifications in the *cyp51A* gene (Hagiwara et al., 2018; Sharma et al., 2019). These *cyp51A*-independent resistant strains have been increasingly documented in certain regions. For instance, data shows that in Japan and the United States, 40% and 60% of azole-resistant isolates, respectively, lack any *cyp51A*-related resistance mechanism (Hagiwara et al., 2018). In contrast, studies in the Netherlands report a lower prevalence, with 15% of azole-resistant isolates showing no *cyp51A* modifications (Buil et al., 2019).

Several genes have been identified as potential contributors to azole resistance in *A. fumigatus*, including ABC transporters such as *cdr1B*, *mdr1*, *mdr2* and *mdr4* (Nywening et al., 2020). Among these, the ABC transporter *Cdr1B* is the only one found to be directly linked to azole resistance (Fraczek et al., 2013). Hmg1, as another key contributor to clinical triazole resistance and is increasingly recognized as a potential factor responsible for azole resistance in *cyp51A*-wild-type ARAF strains (Hagiwara et al., 2018). The *hmg1* gene encodes HMG-CoA reductase (HMGR), an essential enzyme that catalyzes the conversion of HMG-CoA to mevalonic acid, a key rate-limiting step in the ergosterol biosynthesis pathway (Alcazar-Fuoli et al., 2008). Mutations in *hmg1* have been linked to multi-azole resistance, as reported in several studies (Hagiwara et al., 2018; Handelman et al., 2021; Liang et al., 2021; Rybak et al., 2019b; Wu et al., 2020). Most of the amino acid changes identified in Hmg1—such as F262del, S269F, S305P, G307R/D, E307D, P309L, F390Y, and L413P—are located within the sterol-sensing domain (SSD). Among them is S269F mutation that has been linked to increased ergosterol levels in the cell wall, suggesting its role in azole resistance (Hagiwara et al., 2018). This conserved trans membrane motif, anchored to the endoplasmic reticulum, plays a role in regulating the sterol synthesis pathway but does not directly affect the enzyme’s catalytic activity. Additionally, other mutations outside the SSD, like E105K, G466V, S541G, and H564Y, have been identified; however, these mutations are found in both azole-susceptible and azole-resistant strains, suggesting they may not play a significant role in azole resistance (Liang et al., 2021; Takeda et al., 2021; Wu et al., 2020).

Usually, mutations in *hmg1* have been associated with azole resistance as the sole resistance mechanism. However, these mutations often coexist with Cyp51A mutations (Alcazar-Fuoli et al., 2008; Rybak et al., 2019b) and, more recently, with mutations in Cyp51B (Gonzalez-Jimenez et al., 2020).

In the present work, we analyzed nine ARAF isolates, including those with known mutations, novel mutations, and those lacking mutations in the azole target gene *cyp51A*, to identify single nucleotide polymorphisms (SNPs) in *hmg1* that may contribute to azole resistance. The *hmg1* gene was sequenced, and mutational analysis was performed. Furthermore, we investigated the expression of efflux transporter genes to evaluate their potential role in azole resistance.

## 2 Materials and methods

### 2.1 Environmental sampling and isolation of *A. fumigatus*

A total of 156 agricultural soil samples were collected from 13 states across India. The primary focus of sample collection was on agricultural lands, particularly those with documented azole fungicide usage, as these sites are likely to harbor azole-resistant *Aspergillus* species. The number of sample collected from each state is as follows: Andhra Pradesh (n=13), Assam (n=13), Bihar (n=11), Haryana (n=26), Madhya Pradesh (n=6), Punjab (n=9), Rajasthan (n=10), Uttar Pradesh (n=15), and West Bengal (n=17). However, Samples were also collected from regions with relatively lower agricultural activity, such as Delhi (n=9), Himachal Pradesh (n=17), Odisa (n=8), and Uttaranchal (n=2).

All samples were processed and inoculated onto potato dextrose agar (PDA) plates following the method described by Sen et al (Sen et al., 2023). The plates were incubated at 28 ± 2 °C for five days. *Aspergillus* isolates growing on PDA were identified based on their macroscopic and microscopic characteristics, as described by de Hoog et al. (“Atlas of clinical fungi (2nd edn). de Hoog et al., 2001). The identified isolates were then subcultured on PDA and stored at 4 °C for future use.

### 2.2 Antifungal susceptibility testing

The spores (conidia) of all *A. fumigatus* isolates were harvested in sterile phosphate-buffered saline (1× PBS) supplemented with 0.05% Tween 20. The suspension was then adjusted to a concentration of 1×10^4^ conidia/mL in potato dextrose broth. In addition, the susceptible reference strain *A. fumigatus* ATCC 46645 was included as a control in the susceptibility assays. *In vitro* susceptibility testing was conducted to determine the minimum inhibitory concentrations (MICs) of itraconazole (ITC), voriconazole (VRC), and posaconazole (POS) using a slightly modified CLSI M38-A2 broth microdilution method (Alexander and Clinical and Laboratory Standards Institute, 2017) in 96-well flat-bottom polystyrene plates (Tarsons, India). Each isolate was tested in triplicate.

For ITC, VRC, and POS, azole drug stocks were prepared in dimethyl sulfoxide and diluted two fold in a 96-well microplate to reach final concentrations between 64 and 0.125 µg/mL. A 100 µL conidial suspension was added to each well, except for the negative control. The plates were incubated statically at temperature 28 ± 2 °C for 4 days. The results were interpreted based on the proposed epidemiological cutoff values (ECVs) for ITC (1 µg/mL), VRC (1 µg/mL), and POS (0.25 µg/mL). The MIC was defined as the lowest drug concentration with no visible growth compared to the drug-free control (Binder et al., 2019).

### 2.3 Molecular identification of ARAF isolates

Genomic DNA was extracted from the nine ARAF isolates using the cetyl trimethyl ammonium bromide (CTAB) method (Lee et al., 1988; Wu et al., 2001). Molecular identification of the isolates as *A. fumigatus* was confirmed by amplifying and sequencing the full-length 18S internal transcribed spacer (ITS) region using the ITS1 (5’ TCC GTA GGT GAA CCT GCGG 3’) and ITS4 (5’ TCC TCC GCT TAT TGA TATGC 3’) primers (White et al., 1990).

### 2.4 Sequencing of *cyp51A* and *hmg1* gene

Two distinct primer sets, as described by (Tangwattanachuleeporn et al., 2017), were employed to amplify mutations in the *cyp51A* gene (**Supplementary Table 1**, **Supplementary Information**). Additionally, four primer sets were designed and utilized to amplify mutations in the *hmg1* gene. The *hmg1*-specific primers were designed using Primer 3 software (http://primer3.ut.ee/) (Untergasser et al., 2012). The complete list of primer sets used in this study is provided in **Supplementary Table 1**, **Supplementary Information**.

The amplified products were analyzed using agarose gel electrophoresis and purified with the HiMedia Quick Gel Purification Kit following the manufacturer’s instructions. Nucleotide sequencing was performed using Sanger’s sequencing on an ABI 3700 XL (Applied Biosystems). The *cyp51A* and *hmg1* gene sequences were aligned and compared with the wild-type *A. fumigatus cyp51A* sequence (accession no. AF338659) (Lee et al., 2018; Mellado et al., 2007) and *hmg1* sequence (accession no. NC_007195.1), respectively. Sequence alignment was conducted using NCBI BLAST (https://blast.ncbi.nlm.nih.gov/Blast.cgi), and multiple sequence alignment was performed with Clustal Omega (https://www.ebi.ac.uk/Tools/msa/clustalo/).

### 2.5 Homology modelling

Homology models of the Cyp51A protein were generated using MODELLER (version 10.4). The crystal structure (PDB ID: 4YUM) was used as the template based on sequence similarity. Sequence alignment between the template and target sequences was prepared and used as input for modeling.

A total of four models were constructed: three mutant models (A–C), representing different combinations of novel mutations along with known polymorphisms, and one wild-type model (D). The models were generated using the automodel class of MODELLER, which builds protein structures by satisfying spatial restraints derived from the template. Initially, five independent models were generated for each sequence to account for conformational variability arising from stochastic optimization. The generated models were evaluated using the normalized DOPE (Discrete Optimized Protein Energy) scoring function implemented in MODELLER. For each set, the model with the lowest DOPE score was selected as the most reliable structural representation. To ensure reproducibility, a final single model was generated for each sequence using the same alignment and modeling parameters in MODELLER (v10.4), with the number of output models restricted to one. These final models correspond to the best-scoring conformations obtained during the initial modeling step. Structural alignment of the mutant models (A–C) with the wild-type model (D) was performed using PyMOL to assess conformational differences. The root mean square deviation (RMSD) values were calculated to quantify structural variation between the mutant and wild-type proteins.

### 2.6 Molecular docking

The 3D structures of VRC (PubChem ID- 71,616) and ITC (PubChem ID- 55283) were retrieved from the PubChem (https://pubchem.ncbi.nlm.nih.gov/) in Spatial Data File (SDF) format. Using OpenBabel tool, input file of ligands in the SDF file format (.sdf) was converted to the PDB file formats (.pdb) for molecular docking (O’Boyle et al., 2011).

The three-dimensional structures of the mutant Cyp51A models (A–C) generated through homology modeling were used as receptor molecules. The protein molecules were further optimized using AutoDock4 Tool for the molecular docking (Morris et al., 2009). Docking simulations were performed using AutoDock 4.2.3 (Morris et al., 2009). A grid box of size 60 × 60 × 60 Å was defined to encompass the entire protein structure. The docking calculations were carried out using the Lamarckian Genetic Algorithm (LGA), with the number of docking runs set to 50 while keeping other parameters at default values. The resulting docking poses were clustered based on an all-atom root mean square deviation (RMSD) cut-off of 0.3 Å to eliminate redundancy, yielding representative binding conformations. The protein was treated as rigid during the docking process. The best docking poses were selected based on binding energy and cluster size, and interaction analysis was performed using Discovery Studio Visualizer (Şimşek and Duman, 2017).

### 2.7 Quantification of gene expression by qRT-PCR

Quantitative real-time reverse transcription PCR (qRT-PCR) was employed to assess the expression levels of the *cyp51A, cyp51B, hmg1, mdr1, mdr4, mfsC, cdr1B* and *abcA* genes in nine ARAF isolates along with a susceptible *A. fumigatus* (ATCC 46645; control). RNA was extracted from harvested mycelia using TRIzol reagent (Thermo Fisher) (Kamboj et al., 2022). Gene expression was assessed without prior exposure to azole drugs to evaluate baseline expression levels. Inducible expression in response to azole exposure was not assessed and represents a limitation. The extracted RNA was reverse transcribed into first-strand cDNA using the Hi-cDNA synthesis kit (HiMedia, India) according to the manufacturer’s instructions.

Real-time qPCR was then conducted using an ABI QuantStudio 3 (Applied Biosystems, Streetsville, Canada) using SYBR-green master mix (HiMedia), following the protocol described by Gupta et al (Gupta et al., 2022). The thermal cycling conditions consisted of an initial denaturation at 95 °C for 3 min, followed by 40 cycles of 95 °C for 30 s, 60 °C for 30 s, and 72 °C for 30 s. A melt curve analysis was conducted at 95 °C for 15 s, 60 °C for 60 s, and 72 °C for 30 s. Gene expression levels were calculated using the 2^−ΔΔCt^ method, with *β-tubulin* as the reference gene. The primer sets used in this study are listed in **Supplementary Table 1**, **Supplementary Information**.

### 2.8 Statistical analysis

Relative gene expression levels were compared using a one-way ANOVA test. The experiment was performed in biological and technical triplicates, and a *p* value of ≤0.05 was considered statistically significant. All statistical analyses were conducted using GraphPad Prism v8.0.2.263 version and Microsoft Excel.

## 3 Results

### 3.1 Antifungal susceptibility testing

**Figure 1:**
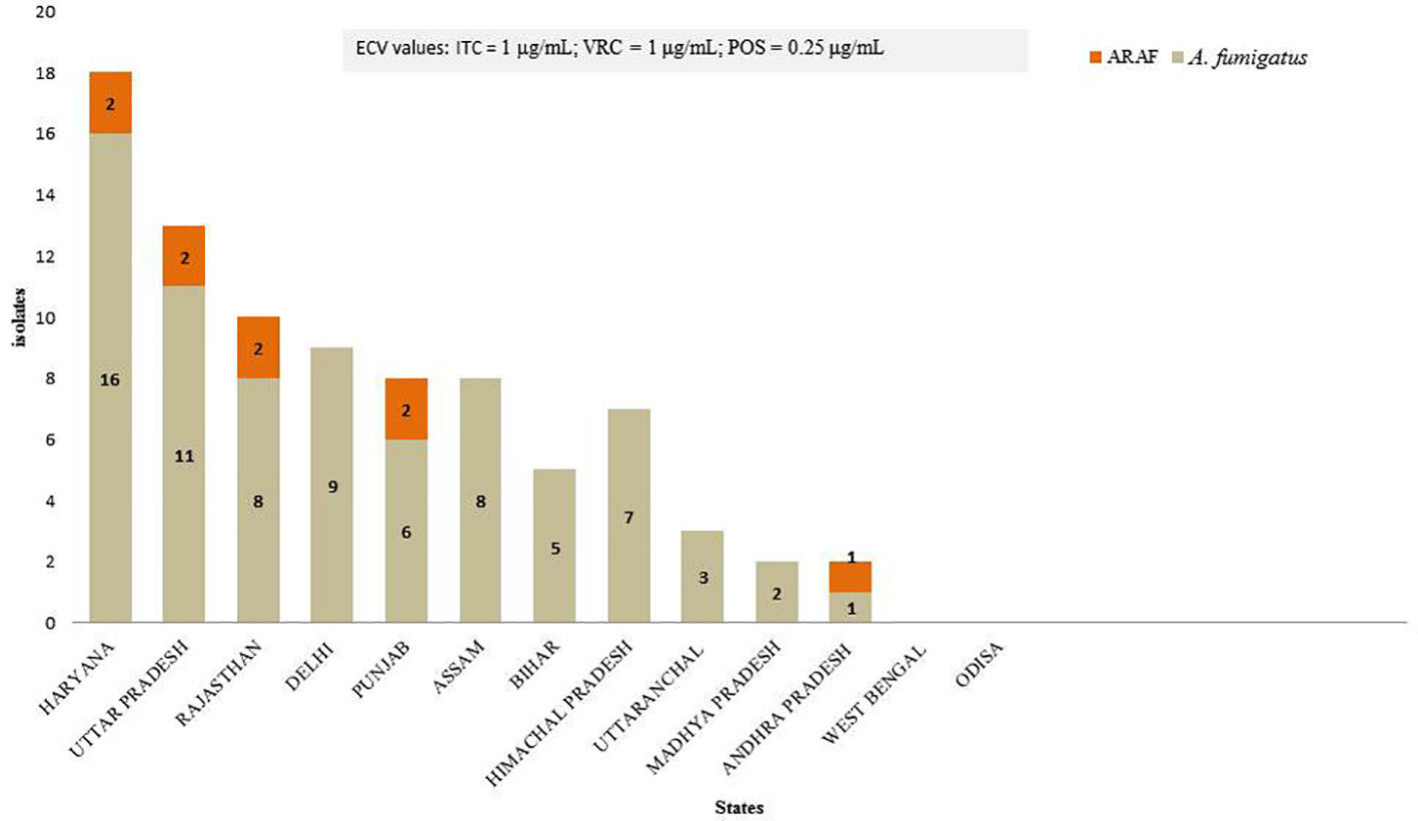
*Aspergillus fumigatus* (n=85) identified from soil samples collected from different states of India. The gray bars represent the number of susceptible isolates, and the red bars represent the number of azole-resistant *Aspergillus fumigatus* isolates. *Aspergillus fumigatus* was classified as resistant (R), when MIC was above the following ECV values: itraconazole >1 μg/mL, voriconazole >1 μg/mL, and posaconazole >0.25 μg/mL.

Antifungal susceptibility analysis against ITC, VRC, and POS was conducted. Azole susceptibility testing revealed that 9 out of 85 A*. fumigatus* isolates were resistant to at least one azole drug. Resistant isolates were obtained from samples collected in Haryana (2/18), Punjab (2/8), Rajasthan (2/10), Uttar Pradesh (2/13), and Andhra Pradesh (1/2) (**Figure 1**). However, the number of resistant isolates was relatively small (n = 9), and the distribution of samples across states was uneven. Therefore, the observed geographic distribution should be considered preliminary and interpreted with caution.

**Figure 2:**
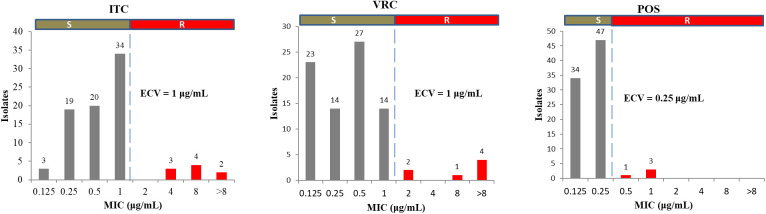
Distribution of *Aspergillus fumigatus* isolates based on the minimum inhibitory concentration (MIC) values (n=85). Vertical dashed lines indicate the epidemiological cutoff values (ECVs) for itraconazole (1 µg/mL), voriconazole (1 µg/mL), and posaconazole (0.25 µg/mL). Isolates with MIC values < ECV were classified as susceptible (S), whereas those with MIC values > ECV were categorized as resistant (R). The gray bars represent the number of susceptible isolates, and the red bars represent the number of azole-resistant *Aspergillus fumigatus* isolates.

Specifically, nine isolates had MIC values above the epidemiological cut of value (ECV) of >1 μg/mL for ITC. Seven isolates had MIC >1 μg/mL for VRC (**Figure 2**) and four isolates had MIC >0.25 μg/mL for POS. Except for two isolates (P17 and RTH30), all the isolates showed cross-resistance for multiple azole drugs (**Table 1**). Four isolates (R2, R5, OFF28, and P01) were cross-resistant to ITC and VRC, While three isolates (UNK2, WDK8, and COI) exhibited cross-resistance to ITC, VRC, and POS drugs.

**Table 1:** Azole susceptibility profiles, *cyp51A* and *hmg1* mutations in azole-resistant *A. fumigatus* isolates.

Isolates	Region (State)	MIC values (µg/mL)			*cyp51A* mutations	*hmg1* mutations
		ITC	VRC	POS		
Known resistance-associated mutations						
UNK2	Haryana	**32**	**32**	**1**	TR46/T289A	G466V, S541G, A648T, Y564H
COI	Andhra Pradesh	**32**	**32**	**1**	TR46/T289A	S541G
WDK8	Uttar Pradesh	**8**	**32**	**1**	TR46/T289A	G446V, S541G, A648T, Y564H, S212P
OFF28	Uttar Pradesh	**8**	**2**	0.25	TR46/Y121F	G466V, S541G, A648T, Y564H
Known polymorphisms						
P01	Punjab	**8**	**2**	0.25	F46Y	D200G, A648T, C669W
R5	Rajasthan	**4**	**8**	0.25	F46Y, M172V, N248T, D255E, E427K	S212P, **G385D**, G466V, S502C
Novel mutations						
P17	Punjab	**4**	0.5	0.25	F46Y, M172V, N248T, D255E, E427K, **L77W**	A648T, K663R, S682N, R692Q
R2	Rajasthan	**8**	**32**	**0.5**	F46Y, M172V, N248T, D255E, E427K, **L477I**	**F399V, T410P**, A648T, K1018N
RTH30	Haryana	**4**	0.125	0.125	F46Y, M172V, **A189P, W223G**	**F362Y,** A648T, C669W, A678T, T661P

*Classification is based on *cyp51A* mutations, which are categorized as known resistance-associated mutations, known polymorphisms, and novel mutations.

Bold MIC values depict MIC above ECV values: > 1 μg/mL for itraconazole and voriconazole and > 0.25 μg/mL for posaconazole. Bolded mutations in the *cyp51A* gene represent novel mutations. Bolded mutations in the *hmg1* gene indicate alterations within the sterol sensing domain.

### 3.2 Molecular identification of ARAF isolates

The 18S ITS region was amplified using PCR and visualized on an agarose gel with ethidium bromide staining. The amplified ITS regions were subsequently sequenced. Sequence analysis revealed that the nine ARAF isolates exhibited 99%–100% identity with *A. fumigatus* sequences in the NCBI database. The identified fungal 18S ITS sequences were submitted to the GenBank NCBI database (https://www.ncbi.nlm.nih.gov/), and GenBank accession numbers (PQ812478, PQ812479, PP784296, PQ812476, PP784298, PP784297, PP784294, PQ812477, and PP784295) were assigned to the submitted ITS sequences.

### 3.3 Mutation analysis of the *cyp51A* gene

The *cyp51A* gene of nine ARAF isolates were sequenced and compared with the susceptible *A. fumigatus* strain (accession no. AF338659) to identify non-synonymous mutations. The analysis revealed amino acid alterations resulting from nucleotide substitutions in the *cyp51A* gene (**Table 1**). The identified mutations were categorized into (i) known resistance-associated mutations, (ii) known polymorphisms, and (iii) novel mutations.

Known resistance-associated mutations were detected in four isolates. Among these, three isolates (UNK2, COI, and WDK8) carried the TR46/T289A mutation, a well-known resistance-associated alteration. These isolates exhibited high MIC values for ITC (8–32 µg/mL), VRC (32 µg/mL), and POS (1 µg/mL), consistent with previously reported high-level resistance associated with tandem repeat insertions. Additionally, isolate OFF28 harbored the TR46/Y121F mutation, another established resistance-associated mechanism. This isolate showed resistance to ITC (8 µg/mL) and VRC (2 µg/mL), while remaining susceptible to POS, further supporting the role of this mutation in conferring azole resistance.

Known polymorphisms, including F46Y, M172V, N248T, D255E, and E427K, were observed in multiple isolates. The F46Y mutation alone was detected in isolate P01, which exhibited resistance to ITC (8 µg/mL) and VRC (2 µg/mL). A combination of all five polymorphisms was found in three isolates (P17, R2, and R5). Despite sharing these polymorphisms, these isolates displayed variable resistance profiles, suggesting that these mutations are not directly associated with azole resistance. Importantly, these mutations have also been widely reported in azole-susceptible isolates in many studies, indicating that they are not involved in conferring azole resistance and are considered neutral polymorphisms.

Novel mutations were identified in three isolates. Isolate P17 harbored the novel mutation L77W in addition to the five known polymorphisms and showed resistance to ITC (4 µg/mL). Isolate R2 contained the novel mutation L477I along with five known polymorphisms and exhibited high-level resistance to ITC (8 µg/mL), VRC (32 µg/mL), and POS (0.5 µg/mL). In isolate RTH30, two novel mutations (A189P and W223G) were detected in combination with two known polymorphisms (F46Y and M172V), and this isolate showed resistance to ITC (4 µg/mL) only. Overall, isolates P17, R2, and RTH30 carrying novel mutations warrant further investigation to elucidate their potential contribution to azole resistance. In contrast, isolates P01 and R5, which harbor only known polymorphisms, may involve alternative resistance mechanisms beyond *cyp51A* alterations.

### 3.4 Homology modeling and molecular docking

**Figure 3:**
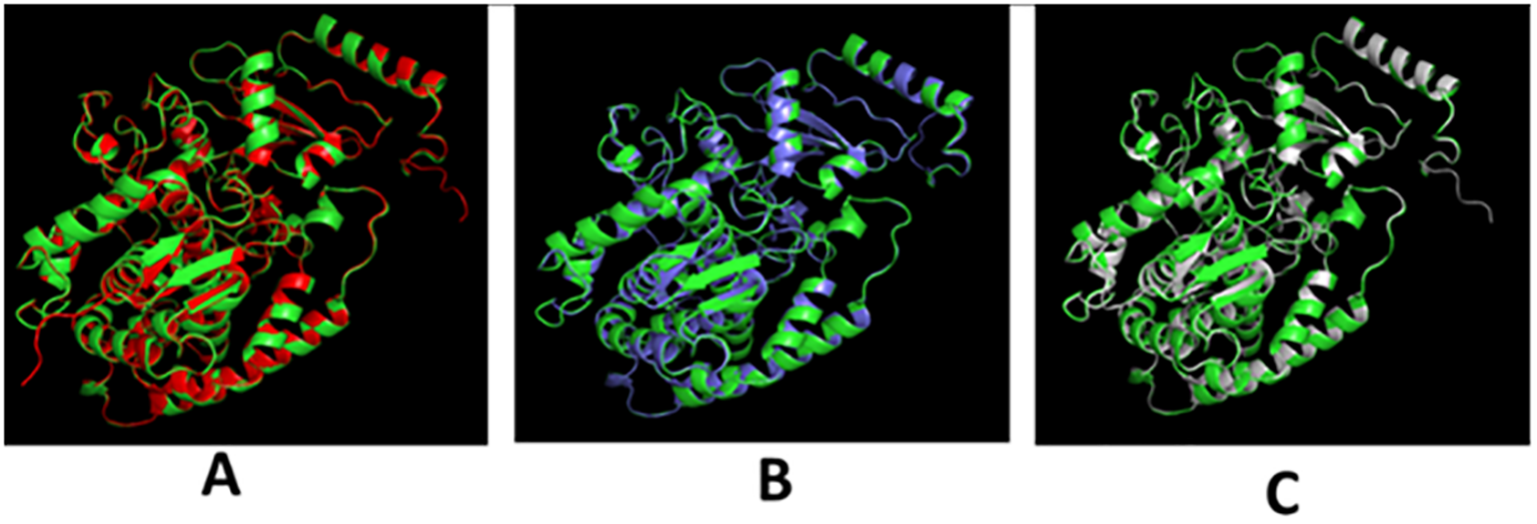
Ribbon representations of superimpositions of modeled **(A-C)** mutated *Cyp51A* protein structure with the wild-type protein of *A. fumigatus.* The green color structure represents the wild-type Cyp51A proteins.

Four homology models (A to D) were constructed for *A. fumigatus.* Models A to C correspond to various novel mutations, in combination with known polymorphism, while model D represents the wild-type protein. These models were aligned with the wild-type Cyp51A protein to assess structural changes (**Figure 3**). Root Mean Square Deviation (RMSD) analysis showed high structural similarity among model A to C compared to the susceptible *A. fumigatus* model D presented in **Table 2**. This indicates that mutations may not involve significant structural disruptions in the protein’s backbone.

**Table 2:** RMSD values of the aligned *Cyp51A* models of the mutated isolates with wild-type *Cyp51A* of *A. fumigatus*.

Mutation	Model	RMSD Score (Å)
F46Y, M172V, N248T, D255E, E427K, **L77W**	A	0.172
F46Y, M172V, N248T, D255E, E427K, **L477I**	B	0.216
F46Y, M172V, **A189P**, **W223G**	C	0.253

*Bold represents novel mutations.

The molecular docking study assessed the binding interactions of ITC and VRC with different *A. fumigatus* Cyp51A protein models (A to C). **Table 3** showed the docking score of the different Cyp51A protein models of the aspergilli isolates with ITC and VRC. Compared to wild-type isolates. Among the models, Model C displayed the highest negative docking scores (-8.21 kcal/mol for VRC). In terms of hydrogen bond interactions, Models A and, C formed 4 hydrogen bonds with VRC. Whereas model B formed 3 H-bonds with VRC.

**Table 3:** Docking scores of the different *Cyp51A* protein models of the *A. fumigatus* isolates with ITC and VRC.

Model	Mutation	Ligand	Docking score (kcal/mol)	H-bond
A	F46Y, M172V, N248T, D255E, E427K, **L77W**	ITC	-8.22	0
		VRC	-7.66	4
B	F46Y, M172V, N248T, D255E, E427K, **L477I**	ITC	-8.56	1
		VRC	-7.66	3
C	F46Y, M172V, **A189P, W223G**	ITC	-8.11	1
		VRC	-8.21	4

*Bold represents novel mutations.

### 3.5 Mutation analysis of the *hmg1* gene

The mutational analysis of the *hmg1* gene revealed a combination of novel and previously reported mutations, with some novel mutations occurring within the SSD of the Hmg1 protein (**Table 1**).

We identified a total of 18 non-synonymous mutations in the *hmg1* gene among the ARAF isolates. Of these, 14 mutations were located outside the SSD region of Hmg1. Four mutations—S541G, G466V, S212P, and Y564H (Gonzalez-Jimenez et al., 2021) had been previously reported mutations. While the remaining 10 mutations were novel. Among the novel mutations, the A648T mutation was the most prevalent, detected in all isolates except COI and R5. Other novel mutations outside the SSD were identified but were less frequent. **Table 1** summarizes the amino acid alterations in the *hmg1* gene.

**Figure 4:**
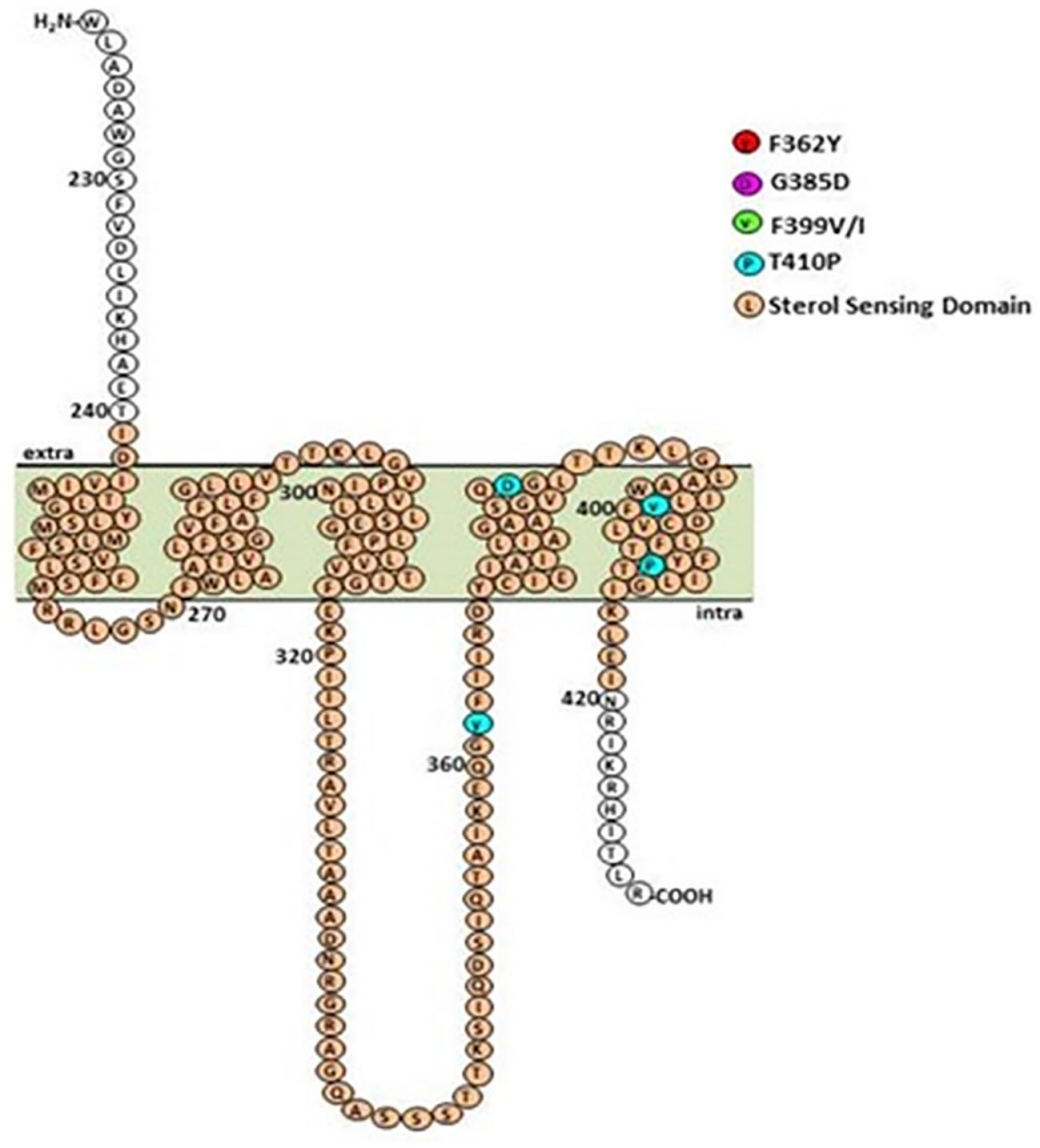
Secondary structure of the sterol sensing domain (SSD) regions of the proteins Hmg1. Brown residues are indicating the SSD with the five transmembrane regions. The amino acids marked in different colors represent the ones in which mutations in SSD regions have been described in this work.

The study also identified a total of four novel mutations within the SSD region, a highly conserved transmembrane domain critical for sterol biosynthesis regulation. Among the nine azole-resistant isolates, three harbored mutations within this domain (**Figure 4**). Specifically, F399V and T410P were detected in isolate R2, G385D in isolate R5, and F362Y in isolate RTH30. As the SSD is a key regulatory domain in sterol biosynthesis, the identified mutations may be associated with altered azole susceptibility in these isolates, and require further functional validation through *in vitro* studies such as CRISPR/Cas9 gene editing, which has previously been applied to *cyp51A* gene in *Aspergillus* species (Umeyama et al., 2018).

### 3.6 Expression analysis of *cyp51A, cyp51B* and *hmg1* genes

**Figure 5:**
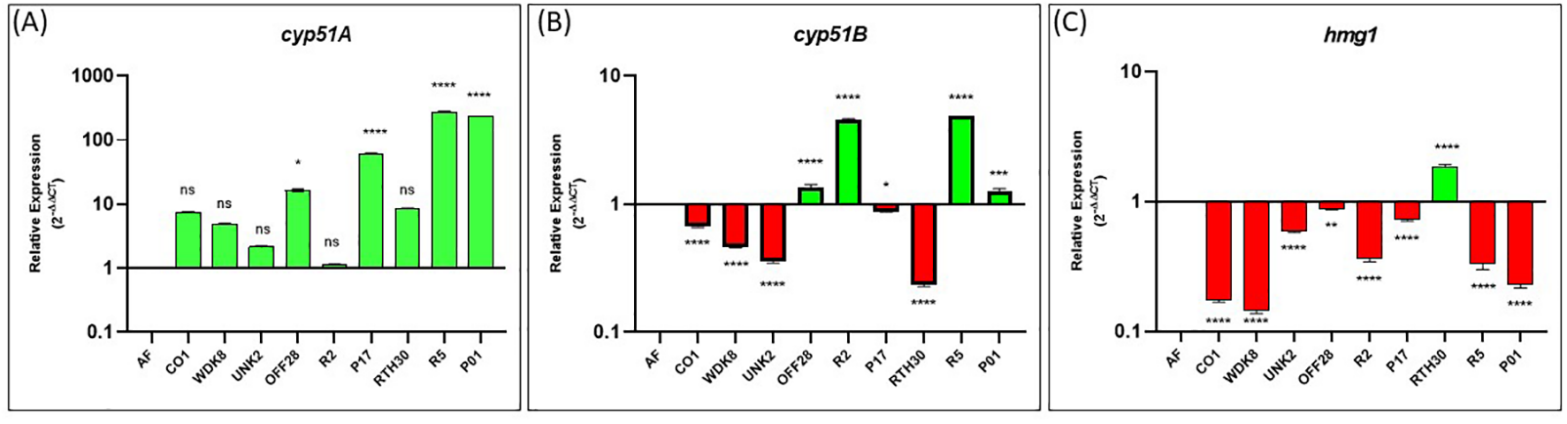
Relative expression of *cyp51A* **(A)**, *cyp51B* **(B)** and *hmg1* **(C)** genes in azole-resistant environmental isolates relative to that in susceptible *A fumigatus* (wild type ATCC 46645). *β-tubulin* was used as a housekeeping gene to normalize transcription levels. Data reported as mean of fold changes with standard deviation from three independent experiments amplified in triplicates. Green color bars represent upregulation while red bars represent downregulation of genes. ****p < 0.0001.

Gene expression of *cyp51A*, *cyp51B*, and *hmg1* was analyzed in ARAF isolates using quantitative qRT-PCR. RNA was reverse-transcribed into cDNA, which was then used as a template for qRT-PCR. Relative expression levels were calculated in comparison with the susceptible *A. fumigatus* strain (ATCC 46645). **Figure 5** indicates the two-fold relative expression of crucial genes in ARAF isolates compared to the susceptible isolate of *A. fumigatus* (ATCC 46645). All isolates exhibited upregulation of the *cyp51A* gene under the tested conditions. Three isolates (P17, R5 and, P01) showed high upregulation (>50 folds) of the gene. Among these R5 and P01 exhibited cross resistance to ITC and VRC, whereas, isolate P17 was resistant for drug ITC only. In contrast, isolate R2, which was resistant to ITC, VRC, and, POS showed only slight upregulation of *cyp51A* (1.3-fold above baseline). Upregulation of the *cyp51B* gene was observed in all three ITC and VRC dual-resistant isolates, and in a ITC, VRC and POS resistant isolate (R2). The *hmg1* gene was downregulated in all the isolates except for the ITC resistant isolate RTH30. In RTH30, the gene exhibited a 1.9-fold upregulation and harbored novel mutation F362Y within the SSD region of *hmg1* gene.

### 3.7 Expression analysis of efflux pumps genes

**Figure 6:**
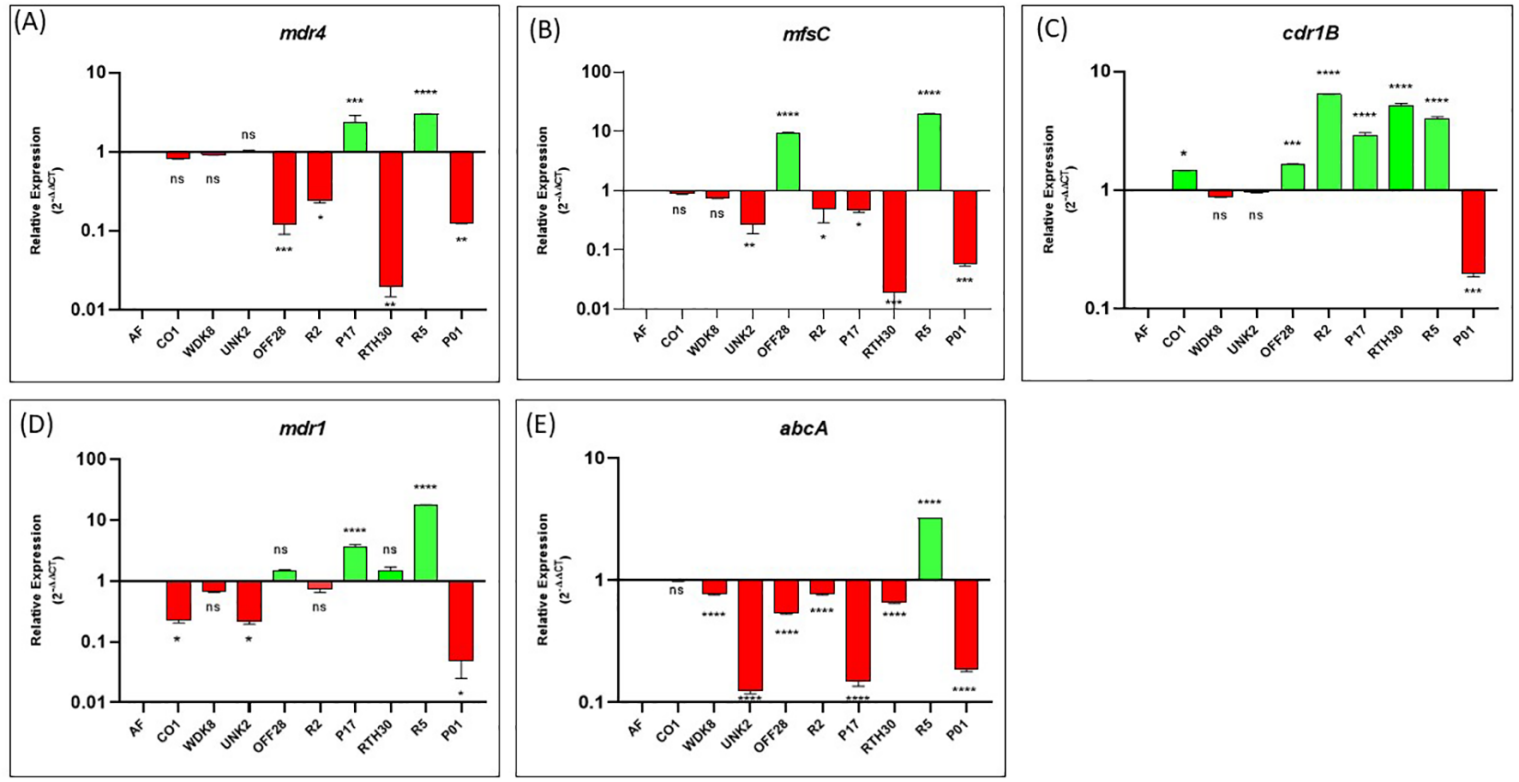
Relative expression of *mdr4* **(A)***, mfsC* **(B)**, *cdr1B* **(C)***, mdr1* **(D)**, and *abcA* **(E)** genes in azole-resistant environmental isolates relative to that in susceptible *A fumigatus* (wild type ATCC 46645) *β-tubulin* was used as a housekeeping gene to normalize transcription levels. Data reported as mean of fold changes with standard deviation from three independent experiments amplified in triplicates. Green color bars represents upregulation while red bars represents downregulation of genes. ****p < 0.0001.

Changes to the expression levels of genes associated with drug efflux pumps (*mdr1, mdr4, mfsC, cdr1B* and *abcA*) were further confirmed. The relative two-fold expression levels of these genes in resistant isolates, compared to a susceptible *A. fumigatus* reference isolate (ATCC 46645), are presented in **Figure 6**.

Among the efflux pump genes, *cdr1B* exhibited the highest frequency of upregulation, with 6 out of 9 azole-resistant isolates showing increased expression. Upregulation was detected in two isolates resistant to ITC (P17 and RTH30), three isolates exhibiting cross-resistance to ITC and VRC (R5, and OFF28), and two isolates (R2 and, COI) resistant to all three azole drugs (ITC, VRC, and POS). Gene *cdr1B* was found to be downregulated in 3 isolates (WDK8, UNK2 and P01). The fold-change expression of *cdr1B* showed considerable variability, ranging from low increases (1.45-fold in COI) to substantial upregulation (6.42-fold in R2). In contrast, *cdr1B* expression was downregulated or remained near baseline levels in three isolates (WDK8, UNK2, and P01). The *mdr1* gene was upregulated in four isolates, including two ITC-resistant isolates (P17 and RTH30) and two ITC and VRC cross-resistant isolates (OFF28 and R5). Expression levels varied widely, with fold changes ranging from low (1.5-fold in OFF28) to markedly elevated expression (17.75-fold in R5).

Gene *mdr4* was upregulated in an ITC resistant isolate P17 (1.9-fold) and in an ITC and VRC cross-resistant isolate R5 (2.98-fold). The gene *mfsC* was upregulated in two ITC and VRC cross-resistant isolates OFF28 (9-fold) and in R5 (19.83-fold). *abcA* gene showed a 3.22-fold upregulation only in an ITC and VRC resistant isolate (R5).

**Figure 7:**
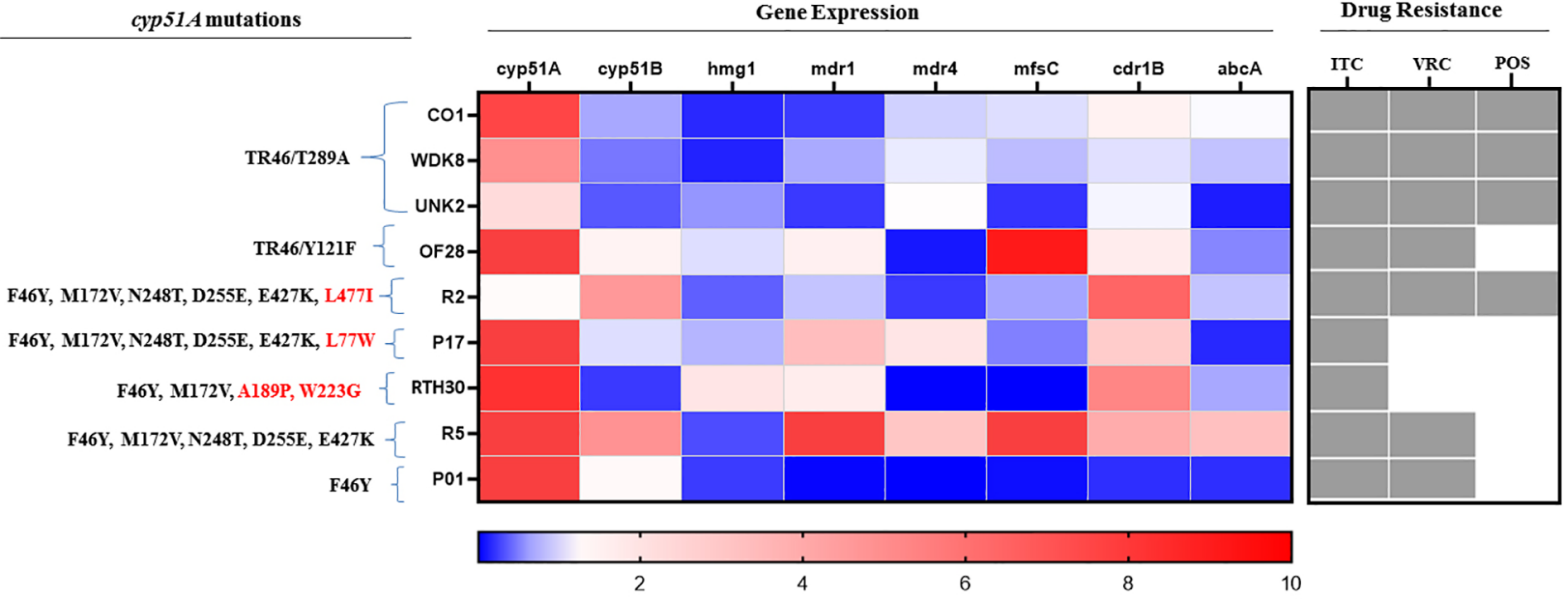
Heatmap of gene expression in ARAF isolates, illustrating the correlation between *cyp51A* mutations, efflux pump gene expression, and azole resistance profiles. The left panel lists the *cyp51A* mutations present in each isolate. Novel mutations are in red letters. The middle panel presents a heatmap showing the expression levels of genes associated with azole resistance (*cyp51A, cyp51B, hmg1, mdr1, mdr4, mfsC, cdr1B*, and *abcA*) across different isolates. The color scale indicates the relative expression, with blue representing downregulation and red representing upregulation compared to the susceptible control isolate (*A. fumigatus* ATCC 46645). The right panel provides an overview of the azole resistance profiles of each isolate, categorized by resistance to itraconazole, voriconazole, and posaconazole. The gray boxes indicate resistance to the corresponding drug, while white boxes indicate susceptibility. This comprehensive overview highlights the complex interplay between genetic mutations and gene expression that contribute to azole resistance in *A. fumigatus*.

Gene expression analysis in ARAF isolates, highlighting the association between *cyp51A* mutations, efflux pump gene expression, and azole resistance profiles, is illustrated in **Figure 7**.

## 4 Discussion

The increasing prevalence of azole-resistant *A. fumigatus*, driven by agricultural fungicide use and clinical azole therapy, poses a significant global health concern. Although environmental azole resistance in *A. fumigatus* has been widely documented worldwide, data from India remain limited (Sen et al 2022).

In the present study, 156 agricultural soil samples from 13 Indian states were analyzed to isolate *A. fumigatus* and determine their susceptibility to azole antifungal agents. 9/85 A*. fumigatus* exhibited resistance to at least one azole drug. Resistant isolates were detected in Haryana (2/18), Punjab (2/8), Rajasthan (2/10), Uttar Pradesh (2/13), and Andhra Pradesh (1/2). Although the number of resistant isolates are limited, the observed distribution aligns with regions known for extensive fungicide application (https://ppqs.gov.in/en/statistical-database), supporting the hypothesis that environmental azole exposure may contribute to the selection of resistant strains. Similar observations have been reported in previous studies that reported azole-resistant *A. fumigatus* isolates in soils exposed to fungicides (Chowdhary et al., 2014a, b). In contrast to previous reports, no azole-resistant isolates were obtained from Bihar or West Bengal, possibly due to the smaller number of samples collected from these states. Broader sampling and long-term monitoring are essential to elucidate the distribution and drivers of azole resistance in Indian agricultural soils.

The most common resistance mechanism *A. fumigatus* involve mutations in the *cyp51A* gene, an increasing number of multi-azole-resistant *A. fumigatus* strains have been identified without *cyp51A* alterations, indicating the emergence of alternative resistance pathways (Rybak et al., 2019a). Studies, including one by Bueid et al (Bueid et al., 2010), have shown that *cyp51A* mutations alone do not account for resistance in approximately 43% of ARAF strains. This highlights the presence of additional, non-*cyp51A* mechanisms contributing to azole resistance. To gain an insight into the role of non-*cyp51A* resistance mechanism of azole resistance, we investigated nine ARAF isolates, including those harbouring known resistance-associated *cyp51A* mutations, known polymorphisms (Mutation not typically linked to resistance), and novel *cyp51A* mutations. Our analysis focused on their azole resistance profiles, specific mutations in the *cyp51A* and *hmg1* genes, and the expression patterns of *cyp, hmg1* and various efflux pump genes. The present study demonstrated that all nine environmental isolates exhibit resistance to one or more azole drugs, with varying levels of azole-resistance. Consistent with other reports, the majority of isolates show resistance between ITC and VRC, with some isolates also resistant to POS. Three isolates (UNK2, WDK8, and COI) demonstrated high-level multi-azole resistance, with resistance observed to ITC, VRC, and POS. These three isolates were found to harbor the TR46/T289A mutation in the *cyp51A* gene, a known determinant of broad-spectrum azole resistance. Another isolate OFF28, which was resistant to both ITC and VRC, harbored the TR46/Y121F mutation in the *cyp51A* gene. The presence of a 46-bp tandem repeat in the *cyp51A* promoter region, combined with the substitutions of tyrosine 121 for phenylalanine and threonine 289 for alanine (TR46/Y121F/T289A), has been shown to result in high levels of VRC resistance in *A. fumigatus* (van der Linden et al., 2013). These four isolates are consistent with previously reported findings, further supporting the established role of TR46-associated mutations in conferring azole resistance. Several studies have documented the presence of ARAF isolates harboring the TR46/Y121F/T289A mutation in environmental samples across different countries, including Iran, the Netherlands, the UK, Tanzania, France, Colombia, India, Germany, and Taiwan (Alvarez-Moreno et al., 2017; Bader et al., 2015; Chowdhary et al., 2014a, b; Hurst et al., 2017; van der Linden et al., 2013; Talbot et al., 2018; Wu et al., 2020). In addition to being detected in environmental isolates, this mutation has also been frequently reported in clinical isolates from countries including, United States Taiwan, France Germany, Netherland, India, Tanzania and Denmark (Chowdhary et al., 2017).

In contrast, several isolates in this study harbored mutations that are classified as known polymorphisms, including F46Y, M172V, N248T, D255E, and E427K. These mutations have been extensively reported in both azole-susceptible and azole-resistant isolates worldwide and are not considered to play a direct role in conferring resistance. In the present study, isolates P01 and R5 contained only these polymorphisms yet exhibited resistance to azole drugs, suggesting that these mutations are likely neutral and not responsible for the observed resistance phenotype, may involve alternative resistance mechanisms beyond *cyp51A* alterations. Similar observations have been reported in previous studies (Chowdhary et al., 2017; Escribano et al., 2021; Snelders et al., 2010), where these polymorphisms were found to have no functional impact on azole susceptibility.

Interestingly four novel mutations (L77W, L477I, A189P, and W223G) were identified in three isolates (P17, R2, and RTH30) in this study. In isolates P17 and R2, the novel mutations L77W and L477I, respectively, were detected in combination with five known polymorphisms, Whereas isolate RTH30 harbored two novel mutations (A189P and W223G) along with F46Y and M172V. These novel mutations have not been reported in previous studies and therefore represent newly identified alterations in the *cyp51A* gene. However, it remains unclear whether these mutations are directly responsible for the observed resistance phenotypes. Therefore, Cyp51A protein modeling was performed to further investigate their potential role in azole resistance. Molecular docking analysis revealed that VRC exhibited the most negative docking score with model C (harboring mutations F46Y, M172V, A189P, and W223G), whereas docking scores for ITC were comparable across all models. The docking results did not indicate any significant alteration in the binding site of the Cyp51A protein due to these amino acid substitutions. Furthermore, structural comparisons based on RMSD values suggested minimal conformational differences between the mutant and wild-type proteins.

These findings indicate that the identified *cyp51A* mutations may not cause substantial changes in the overall protein structure or directly interfere with azole binding. However, indirect effects, such as altered protein stability or interactions with other resistance mechanisms, cannot be excluded. Despite these in silico insights, it is important to acknowledge the limitations of computational models. To confirm the functional impact of these mutations, further *in vitro* studies, such as CRISPR/Cas9-mediated gene editing, are required.

HMG-CoA reductase is a key enzyme that regulates the rate-limiting step in ergosterol biosynthesis. Therefore, alterations in the expression or function of the *hmg1* gene, which encodes this enzyme, can directly influence ergosterol production (Liang et al., 2021). In this study, we identified 18 non-synonymous mutations in *hmg1*, which we categorized based on their location and novelty.

Of the 18 mutations, four were located within the highly conserved SSD: F399V, T410P, G385D, and F362Y. Mutations within the sterol- SSD of the Hmg1 protein have recently been suggested as a potential azole resistance mechanism in strains with wild-type *cyp51A*. Since the SSD domain is responsible for binding to the endoplasmic reticulum membrane and maintaining ergosterol homeostasis, alterations in specific amino acids within this region could impair the enzyme’s function, potentially leading to azole resistance (Rybak et al., 2024). A US based study found that 52% of *A. fumigatus* isolates with wild-type *cyp51A* harbored *hmg1* mutations specifically within the SSD (Siopi et al., 2017). Although initial experiments involving ectopic expression did not establish a clear link between these *hmg1* mutations and azole resistance, more recent research employing CRISPR-Cas9 technology demonstrated that specific mutations, including F262_del, S305P, and I412S, substantially increase triazole MICs (Rybak et al., 2019a). Furthermore, clinical studies from Japan have identified ARAF isolates lacking *cyp51A* mutations but harboring novel *hmg1* mutations within the SSD (Hagiwara et al., 2018). Similar observations have been reported from India and Taiwan, where mutations such as S269F and F390Y in *hmg1* were detected in clinical ARAF isolates without *cyp51A* alterations (Sharma et al., 2019; Wu et al., 2020). The identification of SSD mutations (F399V, T410P, G385D, and F362Y) in our isolates is consistent with these global findings and supports the notion that the SSD region may represent a potential hotspot for resistance-associated alterations. However, the precise functional contribution of these mutations to azole resistance remains to be established and warrants further experimental validation.

In contrast, mutations located outside the SSD region were more numerous and included both previously reported substitutions (S541G, G466V, S212P, and Y564H) and several novel mutations (D200G, A648T, C669W, K663R, S682N, R692Q, S502C, A678T, T661P, and K1018N). The biological significance of these non-SSD mutations is less clear. Some of these mutations, such as S541G, have been reported in both azole-resistant and susceptible isolates, suggesting that they may represent neutral polymorphisms or contribute to resistance only in combination with other genetic alterations (Arai et al., 2021). For instance, previous studies have shown that when the S541G mutation in *hmg1* is combined with *cyp51A* mutations like W273S or TR34/L98H, increased drug resistance was observed (Resendiz-Sharpe et al., 2020).

Overall, while mutations within the SSD are more likely to have a direct functional impact on azole resistance, the role of mutations outside this domain remains uncertain. These findings highlight the complexity of *hmg1*-mediated resistance and underscore the need for further functional validation studies to determine their significance.

Further, to better understand the underlying mechanisms of azole resistance among isolates with different *cyp51A* profiles, including isolates harboring known resistance-associated mutations, known polymorphisms, and novel mutations. We assessed the expression of *cyp51A, cyp51B*, *hmg1* and several efflux pump genes, including *mdr1*, *mdr4*, *mfsC*, *cdr1B*, and *abcA*.

The overexpression of the *cyp51A* gene plays a crucial role in azole resistance in *A. fumigatus* (Arastehfar et al., 2021). In the present study, all isolates showed upregulation of the *cyp51A* gene. However, the fold expression of gene varied considerably across isolates, with certain isolates (R5, P01, and P17) showing markedly higher fold changes, suggesting that high-level overexpression may be associated with resistance, whereas low-level increases may represent baseline variation. Although azole exposure was not part of the experimental design, it is plausible that prior environmental exposure to azole fungicides, particularly in agricultural settings, may have contributed to the selection of isolates with altered *cyp51A* expression. Such environmental pressures have been previously implicated in the emergence of azole resistance in *Aspergillus*. However, this remains a hypothesis, and further studies are required to directly establish this link (Shishodia et al. 2019). Further, the *cyp51B* gene was upregulated in four isolates (OFF28, R2, R5 and P01).

In contrast, expression analysis of the *hmg1* gene showed downregulation in all isolates except RTH30, which harbors F362Y mutation in the SSD region. This pattern suggests that hmg1-mediated resistance, if present, may be driven more by structural or functional alterations of the protein rather than transcriptional regulation. The absence of consistent upregulation among *hmg1*-mutated isolates further indicates that gene expression changes alone may not fully explain its role in azole resistance. This observation is supported by previous findings. For instance, a recent study reported that *hmg1*-mutated strains did not exhibit a consistent pattern of differential gene expression, either under VCZ exposure or in untreated conditions. The authors concluded that mutations in Hmg1 are unlikely to be directly linked to changes in *hmg1* expression or in other genes involved in the ergosterol biosynthesis pathway (Gonzalez-Jimenez et al., 2021).

The overexpression of efflux pumps, ABC transporters, and major facilitator superfamily (MFS) transporters has been extensively studied in *Candida albicans* and *Candida glabrata* (Cannon et al., 2009). Similar studies have emphasized the importance of efflux pump gene overexpression in ARAF as well (Fraczek et al., 2013; da Silva Ferreira et al., 2006). In the current study, the *cdr1B* gene was upregulated in most isolates, except for WDK8, UNK2, and P01. Additionally, *mdr1, mdr4*, and *mfsC* showed variable upregulation across isolates with different resistance profiles, including those harboring novel and known mutations. Specifically,*mdr1* gene was upregulated in two ITC-resistant isolates (P17 and RTH30) that harbor novel mutations (L77W, A189P, and W223G, respectively). *mdr1* was also upregulated in two ITC and VRC cross-resistant isolates (OFF28 and R5). Moreover, *mdr4* was upregulated in the ITC-resistant isolate P17 (with the novel mutation L77W) and in the ITC and VRC-resistant isolate R5 (harboring five known mutations: F46Y, M172V, N248T, D255E, and E427K). The *mfsC* gene was also found to be upregulated in the ITC and VRC cross-resistant isolates OFF28 and R5. These findings suggest that efflux pump expression is isolate-specific, and may act in combination with other resistance mechanisms.

Importantly, all expression analyses were conducted under non-induced conditions, indicating that the observed upregulation likely reflects basal expression differences rather than an immediate response to antifungal exposure. Given the environmental origin of these isolates, it is plausible that prior exposure to agricultural azole fungicides may have exerted selective pressure, favoring strains with constitutive efflux pump activity (Shishodia et al., 2019). However, this remains speculative and requires direct experimental validation.

Several investigations into the molecular mechanisms behind triazole resistance in clinical ARAF isolates have revealed significant upregulation of genes like *atrF*, *mfsC*, and especially *cdr1B* and *atrF* (Fraczek et al., 2013; Paul et al., 2017; Sharma et al., 2019; Slaven et al., 2002; Wu et al., 2020). These observations are reinforced by the fact that heterologous expression of *cdr1B* in *Saccharomyces cerevisiae* lacking *Pdr5*, or deletion of this gene from an ARAF isolate carrying TR34/L98H, leads to a substantial decrease in VRC MIC values (Paul et al., 2017). Furthermore, these efflux pumps have varying substrate specificity, with *cdr1B* demonstrating the broadest range (Esquivel et al., 2020). However, in the absence of functional validation experiments in the present study, such as gene knockout or inhibitor-based assays, the contribution of efflux pumps should be considered putative rather than definitive. Therefore, while the observed overexpression may be associated with azole resistance, further studies are required to confirm their precise role in mediating azole resistance in these environmental isolates.

It is important to note that the number of azole-resistant isolates analyzed in this study was limited, and the sampling across geographic regions was uneven. Therefore, although the data provide useful insights into resistance patterns and associated molecular features, the findings should be considered preliminary and may not fully represent broader geographic trends.

Finally, our findings suggest that additional, yet unidentified molecular mechanisms may contribute to azole resistance, particularly in isolates lacking clear *cyp51A*-mediated resistance. The relatively higher expression of transporter genes and increased number of SNPs observed in environmental isolates compared to clinical strains may reflect adaptation to environmental selective pressures.

Overall, these observations highlight the complexity of azole resistance and underscore the need for more comprehensive studies. Future investigations involving larger and more geographically balanced sampling, along with integrated genomic and functional analyses, will be essential to better understand the underlying resistance mechanisms, particularly in isolates lacking *cyp51A*-mediated resistance.

## Data Availability

The datasets presented in this study can be found in online repositories. The names of the repository/repositories and accession number(s) can be found in the article/**Supplementary Material**.
