# Supplementary material for: Understanding multi-azole resistance in Aspergillus fumigatus: the impact of non-cyp51A mutation and efflux pump overexpression
Source: Front Fungal Biol. 2026 May 28;7:1830942. doi: 10.3389/ffunb.2026.1830942 (PMC13253250; doi:10.3389/ffunb.2026.1830942)
Supplement: Supplementary file 1 [file SupplementaryFile1.docx]

**Understanding multi-azole resistance in Aspergillus fumigatus: The impact of non-cyp51A mutation and efflux pump overexpression**

Pooja Sen^1^, Lokesh Kumar^1^, Jata Shankar^2^ and Pooja Vijayaraghavan^1^*

^1^Amity Institute of Biotechnology, Amity University Uttar Pradesh, Sector-125, NOIDA, Uttar Pradesh, India

^2^Department of Biotechnology and Bioinformatics, Jaypee University of Information Technology, Solan, India

***Corresponding author: Pooja Vijayaraghavan, Ph.D.,** Amity Institute of Biotechnology, Amity University Uttar Pradesh, Sector-125, NOIDA, Uttar Pradesh, India

Email: [vrpooja@amity.edu](mailto:vrpooja@amity.edu)

**Table S1.** Primers used for PCR and qRT-PCR

| **Primer** | **Gene Name** | **Primer sequence (5ʹ-3ʹ)** | **References** |
| --- | --- | --- | --- |
| PCR | *hmg1* (Set1) | F: TGCTCGCCATTTTGTCT  R: CCCGCATGATCCATTT | - |
|  | *hmg1* (Set2) | F2: CTCAACCAGTGCCATTTAC  R2: GAGTTTGATGCAGAGGATG | - |
|  | *hmg1* (Set3) | F3: AGCACCACAAAGTCCATTC  R3: AGGAGTTCGAGCAACAACA | - |
|  | *hmg1* (Set4) | F4: GGCTATGCGTTGGAAAA  R4: GCGACAATTCGAGCAA | - |
| qRT-PCR | *cyp51A* | F: CACGTCAAGTCCCTATCTTC  R: GTTTCAGGGACTCCTTTCTT | (Sen et al. 2023) |
|  | *cyp51B* | F: GGCGTCTTCGATGTATGCAA  R: CGAGCGCGAAGCAGTGTAA | (Sharma et al. 2019) |
|  | *hmg1* | F: TTCAACGCTCACGCATCGAC  R: ACCTAGCATATCAAGCATGGC | - |
|  | *mdr1* | F: GCT TGG TGG AGC GCT TTT AC  R: TCA TGG CCG TCC AGC AA | (Sharma et al. 2019) |
|  | *mdr4* | F: TGG GAC TCG TCA TCT CAA C  R: GGT GTG ACA AAC TGG AGG A | (Sen et al. 2023)2023 |
|  | *mfsC* | F: GGC AGT CCC GTC GCT CT  R: CTT CGA CCT CGC GGA GAA | (Sharma et al. 2019) |
|  | *cdr1B* | F: TTCTCTTCACCTCCACCTTTGC  R: CCGGTCTCGGCGAGTTC | (Sharma et al. 2019) |
|  | *abcA* | F: GTCGATCCCCCGAATATGC  R: CGACGTTCGTGTTTCAGTTCA | (Sharma et al. 2019) |
|  | *β-tubulin* | F: TTG ACC CAG CAG ATG TTC G  R: GGG AAT CCA CTC AAC GAA G | (Hoda et al. 2020) |
